# Mosquito Antimicrobial Peptides: Molecular Diversity, Mechanisms of Action, and Translational Potential

**DOI:** 10.3390/cimb48090856

**Published:** 2026-08-24

**Authors:** Yijie Xiao, Xinhao Li, Huchen Qin, Jinqian Li, Zhenxia Ma, Xiaolong Su, Li Gao, Lianghua Wang

**Affiliations:** Department of Biochemistry and Molecular Biology, College of Basic Medical Sciences, Naval Medical University, Shanghai 200433, China

**Keywords:** mosquitoes, antimicrobial peptides, innate immunity, cecropin, defensin, gambicin, attacin, diptericin, Plasmodium, vector control

## Abstract

Mosquito antimicrobial peptides (AMPs) contribute to innate defense against bacteria, fungi, parasites, and, in some experimental contexts, arboviruses. This narrative review evaluates endogenous mosquito AMP families across Aedes, Anopheles, and Culex species and clearly separates them from heterologous peptides introduced into mosquitoes for transmission-blocking studies. The best-supported endogenous families are cecropins, defensins, gambicin, attacin, and diptericin, although gene repertoires vary among mosquito lineages. Mechanistic evidence is strongest for membrane interaction by individual cecropins and defensins. Evidence for intracellular or redox mechanisms is more limited and peptide- and assay-specific; for example, mitochondrial effects have been demonstrated for *Anopheles albimanus* cecropin 3 in isolated rat cardiac mitochondria, whereas DNA binding and membrane permeabilization have been shown for an *Aedes aegypti* cecropin A derivative in *Pseudomonas aeruginosa*. Scorpine, magainin, and human defensin 5 are not mosquito AMPs and are considered separately as heterologous antiplasmodial effectors. Toll and immune deficiency (IMD) pathways regulate mosquito immune genes through NF-κB-family transcription factors, whereas Janus kinase–signal transducer and activator of transcription (JAK–STAT) is a distinct cytokine-signaling pathway. Translational approaches—including transgenic expression, paratransgenesis, *Wolbachia*-based control, and peptide engineering—remain promising but require cautious interpretation because efficacy, mechanism, ecological safety, horizontal gene transfer, regulatory oversight, and durability have not been resolved uniformly. By distinguishing direct peptide activity from genetic, expression-only, and inferential evidence, this review provides a more rigorous framework for evaluating mosquito AMPs and their potential use in vector-borne disease control.

## 1. Introduction

### 1.1. Global Burden of Mosquito-Borne Diseases

Mosquito-borne infections remain a major and evolving public-health challenge. The World Health Organization (WHO) estimated 282 million malaria cases and 610,000 malaria deaths in 2024 [1]. Dengue incidence has also increased markedly: the WHO reports an estimated 100–400 million infections each year and 14.6 million reported cases in 2024 [2]. These estimates should not be combined as though they were generated by a single surveillance system, but together they illustrate the scale of disease caused by pathogens transmitted principally by Anopheles, Aedes, and Culex mosquitoes.

Vector control remains essential, yet its effectiveness is threatened by insecticide resistance, heterogeneous intervention coverage, urbanization, and environmental change. The WHO guidance describes widespread and increasing resistance in malaria vectors and recommends resistance monitoring and deployment of interventions appropriate to local susceptibility profiles [3]. These limitations motivate complementary approaches that target mosquito physiology, immunity, microbiota, or vector competence rather than relying exclusively on conventional insecticides.

### 1.2. Scope

Mosquitoes rely on cellular and humoral innate defenses, including phagocytosis, melanization, complement-like factors, reactive intermediates, and antimicrobial peptides (AMPs). AMPs are produced systemically by the fat body and locally at barrier tissues, but their basal abundance and inducibility vary across tissues, developmental stages, microbial exposures, and mosquito species [4,5,6,7]. This biological heterogeneity is important: detection of an AMP transcript after infection does not by itself demonstrate that the encoded peptide directly kills the challenging organism.

This article focuses on endogenous mosquito AMPs with direct biochemical, genetic, or immune-regulation evidence and on heterologous peptides that have been evaluated as transmission-blocking effectors in mosquito systems. Evidence is described at the level of the peptide, mosquito species, target organism, concentration, and assay whenever those data are available. Established observations are separated from proposed mechanisms, and endogenous mosquito AMPs are distinguished from heterologous peptides delivered by injection, transgenesis, or engineered symbionts.

As a narrative review, this article draws on representative primary studies of mosquito AMP classification, structure, activity, immune regulation, and translational delivery, together with authoritative public-health sources for epidemiological context. To facilitate comparison across heterogeneous studies, the discussion distinguishes direct peptide activity from functional genetic or whole-organism evidence and from expression-only associations, while retaining quantitative activity measures in their original assay-specific units.

## 2. Classification and Diversity of Endogenous Mosquito AMPs

The endogenous AMP repertoire is lineage-specific rather than a fixed set shared by every mosquito. Five commonly described families in *Aedes aegypti* are defensins, cecropins, gambicin, attacin, and diptericin [7]. *Anopheles gambiae* contains multiple cecropin and defensin genes together with gambicin and an attacin-like gene, whereas more recent transcriptomic studies have identified additional predicted effectors in particular species or annotations [4,6,8]. Holotricin-like sequences have also been reported in *Ae. aegypti* transcriptomic datasets [9]. Accordingly, the phrase “five major families” is used here as an operational summary for well-described Aedes AMPs, not as a universal taxonomic rule.

No reliable primary evidence was identified to support hypenducin, hyptextensin, or Hyp as established mosquito AMP families; these terms are therefore not used in the present classification. Likewise, scorpine, magainin, and human defensin 5 are excluded from the endogenous classification. They are biologically relevant to mosquito-based transmission-blocking experiments but originate from scorpion venom, amphibian skin, and humans.

This classification provides a practical framework for the sections that follow, but family membership should be viewed as a starting point rather than a complete predictor of function. Mature-peptide length, charge, amphipathicity, disulfide connectivity, proteolytic processing, and local concentration can all shape activity, while mosquito species and target organism determine the physiological context in which these properties are expressed.

### 2.1. Cecropins

Cecropins are short, usually cationic, cysteine-free peptides that adopt amphipathic α-helical conformations in membrane-mimetic environments. Their precursors contain a signal peptide, and mature mosquito cecropins are generally approximately 34–40 amino acids long. The first purified *Ae. aegypti* cecropin A comprised 34 residues and differed from several lepidopteran cecropins by lacking tryptophan and C-terminal amidation [10]. Cecropin copy number and inducibility differ among mosquito species; therefore, activity reported for one paralog should not automatically be assigned to all cecropins.

Direct antibacterial evidence is available for selected mosquito cecropins. A synthetic 36-residue derivative of *Ae. aegypti* cecropin A, cecropin A2, inhibited clinical *Pseudomonas aeruginosa* isolates at minimum inhibitory concentrations (MICs) of 32–64 µg/mL and other tested Gram-negative bacteria at 2–32 µg/mL [11]. *An. gambiae* synthetic cecropins displayed activity against Gram-positive and Gram-negative bacteria, filamentous fungi, and yeast, although potencies differed by organism [12]. Aedesin, a cecropin-like peptide induced in dengue virus (DENV)-infected *Ae. aegypti* salivary glands, showed antibacterial and antiviral activity in vitro [13,14]. These results support broad potential within the family while also demonstrating why quantitative, peptide-specific reporting is necessary.

### 2.2. Defensins

Mosquito defensins are cysteine-rich peptides with the characteristic insect defensin cysteine-stabilized α/β fold. Mature peptides are typically approximately 40 amino acids long and contain six conserved cysteines forming three disulfide bonds. Multiple defensin paralogs occur in *Ae. aegypti*, and defensin expression is both tissue- and stage-dependent [15,16]. Structural similarity does not guarantee identical target spectra, because charge distribution, surface loops, and target-membrane composition influence activity.

The activity spectrum of recombinant *An. gambiae* defensin 1 (DEF1) is particularly well defined. Most tested Gram-positive bacteria were sensitive at 0.1–0.75 µM; no activity was detected against most Gram-negative bacteria, except selected *Escherichia coli* strains, and growth inhibition was observed against some filamentous fungi but not yeast [17]. In vivo RNA-interference experiments further showed that *An. gambiae* defensin was required for resistance to Gram-positive bacteria but was not a major determinant of *P. berghei* midgut-stage development under the tested conditions [18]. These findings argue against describing mosquito defensins generically as broad-spectrum antiplasmodial peptides.

### 2.3. Gambicin

Gambicin was initially isolated from an *An. gambiae* immune-responsive cell culture. Its precursor is processed to a 61-residue, 6.8 kDa mature peptide containing eight cysteines and four disulfide bridges [19]. Purified gambicin killed both Gram-positive and Gram-negative bacteria, altered the morphology of a filamentous fungus, and was only marginally lethal to cultured *P. berghei* ookinetes [19]. Thus, gambicin is a mosquito-associated AMP with direct activity against several target classes, but the original evidence does not support describing it as uniquely or strongly antiplasmodial.

Gambicin orthologs are present beyond *An. gambiae*, including *Ae. aegypti*, where promoter analysis in Aag2 cells demonstrated combinatorial regulation by Rel1, Rel2, and STAT [7]. This result supports pathway integration at the gambicin promoter in that cell system; it should not be generalized to every mosquito tissue, pathogen, or AMP gene.

### 2.4. Attacin, Diptericin, and Other Glycine-Rich Effectors

Attacins and diptericins are glycine-rich insect immune effectors that are distinct families, although they are evolutionarily related. *Ae. aegypti* genome-based inventories include one attacin and one diptericin in addition to cecropins, defensins, and gambicin [7]. Attacins from non-mosquito insects are best known for activity against Gram-negative bacteria and effects on the outer membrane, but mosquito-specific biochemical characterization remains comparatively sparse [20]. For mosquito attacin and diptericin, much of the available evidence concerns transcriptional induction or pathway regulation rather than MIC values obtained with purified mature peptides [7,9]. Their inclusion as endogenous mosquito AMPs is therefore justified by sequence and immune-regulation evidence, while precise activity spectra remain a knowledge gap.

Holotricin-like transcripts and other predicted AMPs have been reported in *Ae. aegypti* datasets [9], but nomenclature and functional validation remain less mature than for cecropin, defensin, or gambicin. Future classifications should identify the genome build, gene identifier, mature-peptide prediction, and experimental validation status rather than treating every computationally annotated immune peptide as an established family.

### 2.5. Representative Quantitative and Functional Evidence

Table 1 intentionally combines quantitative biochemical data with explicitly labeled functional observations. MIC, half-maximal inhibitory concentration (IC50), and half-maximal effective dose (ED50) values are not interchangeable, and assay systems differ substantially. The table should therefore be used to locate direct evidence, not to rank peptide families across organisms.

## 3. Membrane-Targeting Mechanisms

### 3.1. General Models and Their Evidentiary Limits

**Figure 1:**
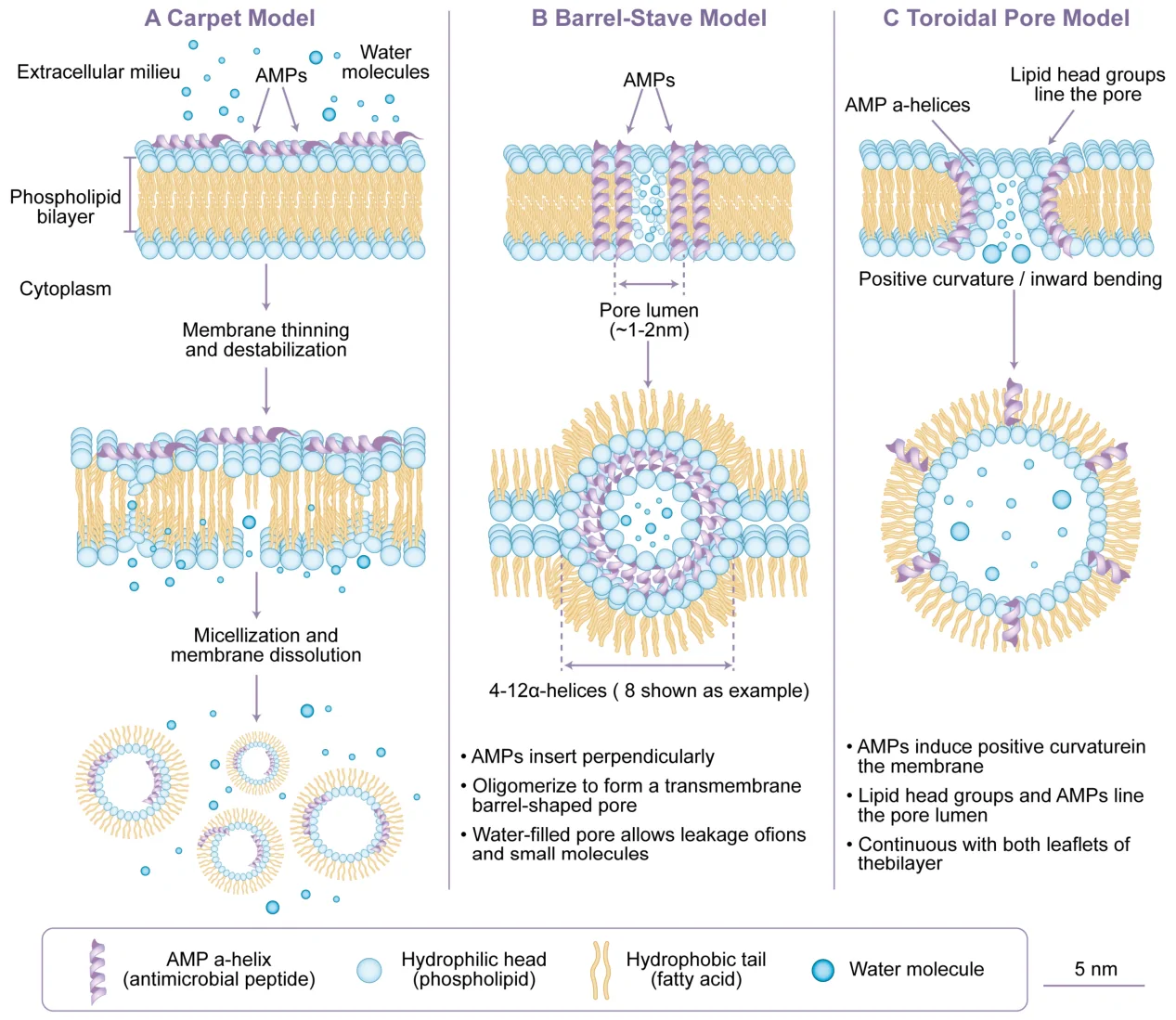
Three conceptual mechanistic models of antimicrobial peptide (AMP)-mediated membrane disruption. AMPs permeabilize microbial membranes through three principal mechanisms that differ in peptide orientation, oligomeric state, and the fate of the lipid bilayer. (**A**) The carpet model depicts AMPs lying parallel to the membrane surface and accumulating to a threshold concentration that thins and destabilizes the bilayer, ultimately leading to micellization and membrane dissolution in a detergent-like manner. (**B**) The barrel-stave model shows AMPs inserting vertically into the bilayer, where 4–12 helices oligomerize to form a transmembrane pore whose hydrophilic faces line the aqueous lumen and whose hydrophobic faces contact the lipid acyl chains. (**C**) The toroidal pore model illustrates AMPs inducing positive membrane curvature such that both peptide helices and lipid headgroups line the pore lumen, producing a continuous structure between the two leaflets. Color key: AMP α-helix (purple); phospholipid hydrophilic head (light blue); hydrophobic fatty-acid tail (pale yellow); water molecules (light-blue circles). Scale bar: 5 nm.

Three conceptual models are commonly used to describe membrane permeabilization by amphipathic AMPs: the carpet, barrel-stave, and toroidal-pore models (Figure 1) [27,28]. In the carpet model, peptides accumulate approximately parallel to the bilayer surface and destabilize it after reaching a threshold surface density. In the barrel-stave model, peptide helices form the wall of a transmembrane aqueous pore. In the toroidal-pore model, peptides and continuously curved lipid monolayers jointly line the pore. These models are useful mechanistic frameworks, but a schematic does not establish which model applies to a particular mosquito peptide in a native pathogen membrane.

### 3.2. Evidence from Individual Mosquito Cecropins

For *Ae. aegypti* cecropin A2, several observations support a multi-step antibacterial mechanism against *P. aeruginosa*. The peptide bound lipopolysaccharide, increased membrane permeability in a concentration-dependent assay, and interacted with bacterial genomic DNA [11]. The MIC against strain PA14 was 32 µg/mL, and experiments at or above the MIC showed rapid permeability changes. These results support membrane damage plus intracellular access for this peptide-target combination; they do not prove a stable barrel-stave pore or establish a family-wide mechanism.

Cecropin conformation and membrane behavior depend on lipid composition, peptide-to-lipid ratio, salt concentration, and peptide sequence [27,28,29]. Consequently, claims about pore size, oligomer number, or orientation require direct structural or electrophysiological measurements for the specific peptide and membrane system. For most mosquito cecropins, the available evidence supports membrane interaction and permeabilization more strongly than it supports a unique named pore architecture.

### 3.3. Defensin Membrane Interactions

Mosquito defensins clearly inhibit susceptible bacteria, but their molecular targets remain incompletely resolved. The *An. gambiae* DEF1 study established target spectrum, killing kinetics, and native peptide induction but did not demonstrate a universal voltage-gated channel mechanism, lipid-II binding, or a specific pore geometry [17]. These mechanisms have been described for selected defensins from other organisms, yet extrapolation across divergent defensin sequences is not warranted. For mosquito defensins, the safest conclusion is that the conserved disulfide-stabilized fold supports interaction with susceptible microbial surfaces, while the decisive molecular target remains peptide- and organism-specific.

## 4. Intracellular, Regulatory, and Redox Effects

### 4.1. Mitochondrial Effects: Direct Observation in a Non-Microbial System

The most direct mitochondrial evidence for a mosquito AMP concerns synthetic *An. albimanus* cecropin 3 tested on isolated rat cardiac mitochondria and in rats [30]. At nanomolar concentrations, the peptide uncoupled oxidative phosphorylation, altered oxygen consumption and calcium transport, increased reactive oxygen species (ROS), inhibited superoxide dismutase, and promoted release of the pro-apoptotic protein Bax. This is a clearly defined experimental observation, but it involves mammalian mitochondria rather than a microbial or Plasmodium target. It therefore demonstrates potential mitochondrial bioactivity and possible host toxicity, not a general antimicrobial mechanism of mosquito cecropins.

No equivalent evidence was identified showing that mosquito defensins, gambicin, or attacin routinely localize to pathogen mitochondria. Statements about mitochondrial disruption should consequently specify cecropin 3, the rat cardiac-mitochondrial system, the nanomolar exposure range, and the biochemical endpoints measured [30]. Whether similar effects contribute to pathogen killing in mosquitoes remains an open question.

### 4.2. Nucleic-Acid Binding and Non-Antimicrobial Nuclear Functions

Cecropin A2 bound purified *P. aeruginosa* genomic DNA in electrophoretic assays while also permeabilizing the bacterial envelope [11]. DNA interaction is therefore plausible after cytoplasmic entry, although its quantitative contribution to bacterial killing was not isolated from membrane damage. By contrast, *Ae. aegypti* cecropin B was detected in pupal-cell nuclei and bound a motif in the AaPPO3 regulatory region, promoting prophenoloxidase expression and normal cuticle formation [31]. This is a developmental gene-regulatory function, not evidence that cecropin B kills microbes by inhibiting transcription or translation.

Available mosquito-specific evidence is insufficient to conclude that cecropins, defensins, gambicin, and attacins generally inhibit ribosomes, protein synthesis, DNA replication, or RNA transcription. Such mechanisms are established for some non-mosquito AMPs, but they should be presented as hypotheses for mosquito peptides until direct target-engagement and loss-of-function experiments are available.

### 4.3. ROS and Redox Context

AMPs operate within a redox-active immune environment, but association should not be confused with a direct peptide mechanism. Cecropin 3 directly increased ROS in isolated rat cardiac mitochondria [30]. Separately, *Wolbachia* infection of *Ae. aegypti* increased host ROS, activated Toll signaling, and elevated several immune genes, including cecropins and defensins [32]. The latter study supports a host signaling cascade—*Wolbachia* to ROS to Toll-associated immune activation—rather than direct ROS production by each AMP.

The mosquito midgut also experiences oxidative stress after blood feeding and infection, and ROS can act independently or in concert with immune effectors. At present, evidence that endogenous mosquito AMPs act as physiologically important antioxidants or ROS scavengers is limited. Proposed redox functions should be tested using purified peptides, defined targets, physiological concentrations, and appropriate ROS-specific controls.

## 5. Pathogen-Dependent Evidence

Pathogen class is only one source of variation. Cell-wall architecture, membrane lipid composition, parasite developmental stage, tissue compartment, ionic conditions, and peptide availability can each alter the apparent phenotype. For this reason, direct biochemical activity, infection-associated expression, and genetic effects in the mosquito should be read as complementary but non-equivalent forms of evidence.

**Figure 2:**
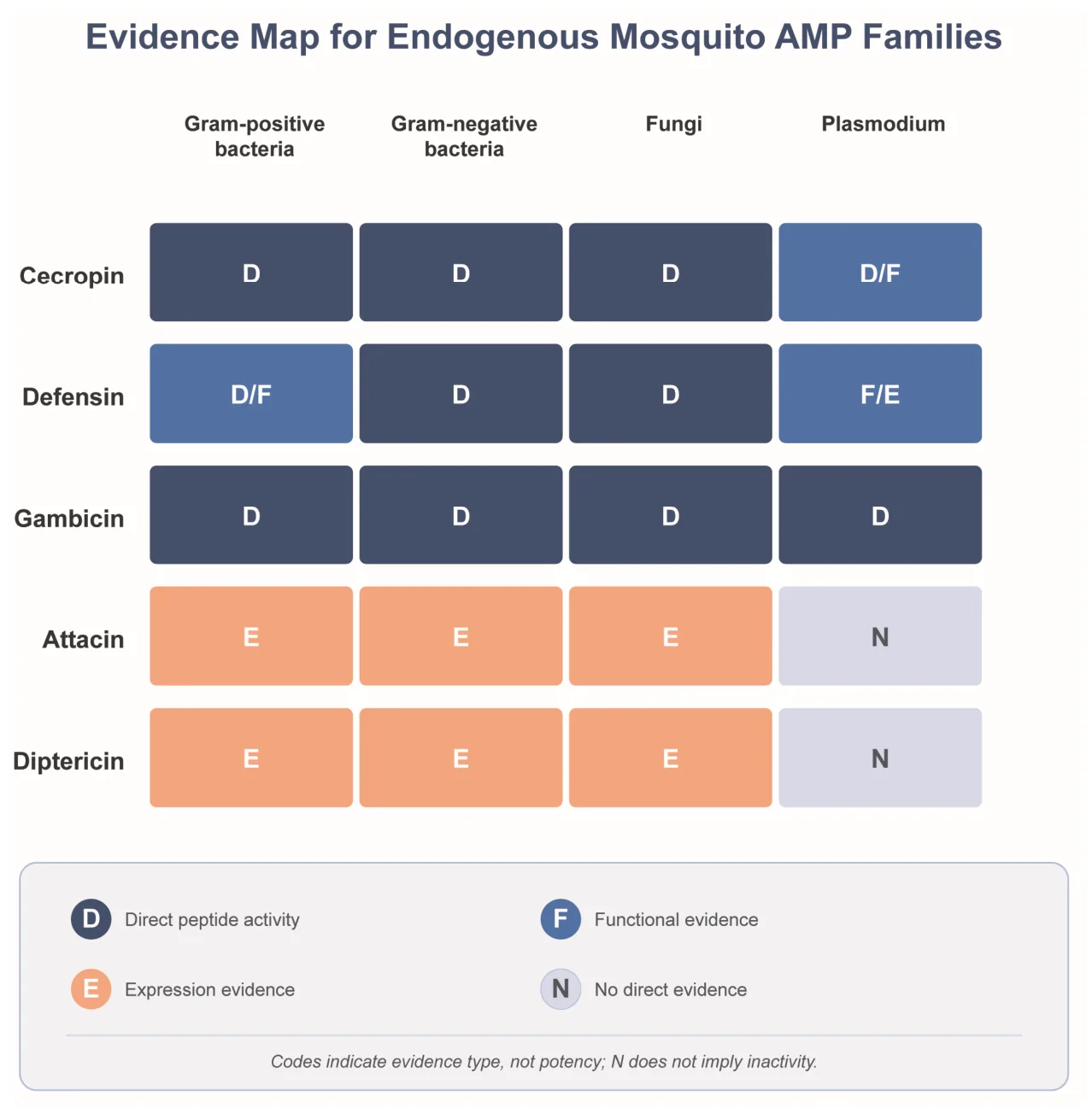
Evidence map for representative endogenous mosquito AMP families and four pathogen classes. D, direct activity of a purified or synthetic mosquito peptide against the indicated target; F, functional whole-organism or genetic evidence, including transgenic expression or gene perturbation; E, expression or induction associated with exposure, without direct demonstration that the peptide mediates target killing; N, no direct functional evidence identified in the studies evaluated. A cell can contain more than one code when evidence types coexist. These categories describe evidence type, not potency, and do not imply a common MIC threshold across assays [7,9,10,11,12,13,14,15,16,17,18,19,21,22,23,33,34].

Because assays differ in peptide preparation, target strain, medium, endpoint, and exposure time, activity cannot be summarized reliably by assigning an entire AMP family a universal label of “strong,” “moderate,” or “weak.” Figure 2 instead classifies the type of evidence available for representative mosquito AMP families. “No direct evidence identified” means that this review did not find a functional test for that family-target combination; it does not mean that the peptide has been proven inactive.

### 5.1. Bacteria

Direct antibacterial evidence is strongest for individual cecropins, defensins, and gambicin. *Ae. aegypti* cecropin A2 inhibited *P. aeruginosa* and other Gram-negative bacteria with organism-dependent MICs [11]. *An. gambiae* DEF1 showed submicromolar activity against most tested Gram-positive bacteria but little activity against Gram-negative species [17]. Purified gambicin killed representatives of both Gram classes [19]. These differences demonstrate functional specialization at the peptide level.

In vivo and transcriptional studies provide complementary information. *An. gambiae* defensin knockdown increased susceptibility to Gram-positive bacterial infection [18], whereas comparative infection studies showed stronger induction of cecropin and defensin transcripts in *Ae. aegypti* than in *An. gambiae* under the tested conditions [16]. In *Ae. aegypti* Aag2 cells, most AMP induction after Gram-negative bacterial challenge depended on the immune deficiency (IMD) pathway [7]. These findings connect AMP regulation to bacterial defense but should not be converted into MIC-like potency statements.

Cecropin A2 and tetracycline reduced each other’s MIC against *P. aeruginosa* eightfold and improved survival in an infected *Galleria mellonella* model [11]. This is evidence of synergy between one mosquito-derived peptide and an antibiotic in a non-mosquito in vivo model. Direct synergy among multiple endogenous mosquito AMPs remains less thoroughly quantified and requires checkerboard, time–kill, or genetic interaction experiments.

### 5.2. Plasmodium: Endogenous Mosquito AMPs

Endogenous mosquito AMPs can affect Plasmodium in specific experimental settings. Immune activation and cecropin expression have long been associated with altered sporogonic development in Anopheles mosquitoes [12,33]. Co-expression of *Ae. aegypti* cecropin A and defensin A reduced *P. gallinaceum* infection, providing proof of principle that engineered AMP combinations can influence parasite development [34]. However, this transgenic experiment does not establish the effect of either peptide alone at its native expression level.

Purified *An. gambiae* gambicin was only marginally lethal to cultured *P. berghei* ookinetes [19], whereas defensin knockdown did not significantly alter *P. berghei* development in *An. gambiae* in one study [18]. More recently, synthetic Anopheles cecropin B inhibited cultured blood-stage *P. falciparum*, with IC50 values of 9.89–12.81 µM across drug-sensitive and drug-resistant strains, but full-length peptide showed cytotoxicity and weak efficacy in a mouse malaria model [21]. Because the latter assays concern vertebrate blood stages rather than sporogonic stages in mosquitoes, their principal relevance is peptide-template development rather than native vector immunity.

### 5.3. Heterologous Antiplasmodial Peptides Tested in Mosquito Systems

Scorpine is a 75-residue peptide isolated from *Pandinus imperator* scorpion venom. It inhibited *P. berghei* ookinetes and gametes with ED50 values of 0.7 and 10 µM, respectively [22]. Engineered *Asaia* strains that secrete scorpine-containing fusion proteins reduced *P. berghei* oocyst burdens in Anopheles mosquitoes [23,24]. Scorpine is therefore a potent heterologous effector for paratransgenesis, not an endogenous mosquito AMP family.

Magainins originate from amphibian skin, and early injection experiments showed that magainins and lepidopteran cecropins disrupted Plasmodium sporogonic development in anopheline mosquitoes [25]. Human defensin 5 is another heterologous peptide: microinjection at 200 µg/mL reduced *P. yoelii* infection in *An. stephensi* and increased expression of Toll-associated genes; silencing MyD88 weakened the protective effect [26]. These studies are relevant to engineered delivery and immune stimulation but should not be used to define the natural mosquito AMP repertoire.

### 5.4. Fungi

Evidence for antifungal activity includes both direct peptide assays and mosquito infection studies. Recombinant *An. gambiae* DEF1 inhibited selected filamentous fungi but not yeast [17], and purified gambicin caused a morphogenic effect on a filamentous fungus [19]. Synthetic *An. gambiae* cecropins also showed activity against filamentous fungi and yeast in vitro [12]. These results are peptide- and species-specific rather than proof that every member of the corresponding family is antifungal.

Entomopathogenic fungal infection produces time-, tissue-, and fungal-strain-dependent changes in mosquito AMP transcription [9,35]. In *Ae. aegypti*, simultaneous silencing of multiple AMP genes altered susceptibility to entomopathogenic fungi more consistently than some single-gene perturbations, supporting combinatorial defense [9]. Environmental determinants of fungal-conidium stability, including UV-B exposure, fall outside the present focus because they do not constitute direct mechanisms of mosquito AMPs. A remaining knowledge gap is the concentration and localization of mature AMP peptides at fungal infection sites in vivo.

### 5.5. Arboviruses

RNA interference (RNAi) is a central antiviral defense in mosquitoes, and AMP responses represent one component of a broader network rather than the dominant universal antiviral mechanism [36]. Aedesin provides direct peptide-level evidence: synthetic mature and precursor-containing forms showed anti-DENV and anti-chikungunya activity in cell-based assays [13,14]. The Ras/ERK pathway has also been linked genetically to AMP-dependent restriction of DENV in Aedes mosquitoes [37]. These studies support antiviral roles for specific peptides and contexts.

*Wolbachia*-infected *Ae. aegypti* can show ROS-dependent Toll activation and increased cecropin and defensin expression; RNAi depletion of selected AMPs weakened DENV restriction in that laboratory system [32]. Nevertheless, *Wolbachia*-mediated pathogen blocking is multifactorial and can involve cellular resource competition, lipid metabolism, immune priming, tissue distribution, and *Wolbachia* density. Field effectiveness of *Wolbachia* releases therefore does not demonstrate that increased AMP expression is the principal causal mechanism.

## 6. Regulation of Mosquito AMP Expression

AMP function in vivo depends not only on intrinsic peptide activity but also on when, where, and to what concentration the peptide is produced. Signaling pathways integrate microbial recognition with tissue state and physiological cues, thereby determining whether an AMP reaches a biologically relevant compartment at the appropriate stage of infection.

### 6.1. Toll and IMD Are NF-κB Pathways; JAK–STAT Is Distinct

**Figure 3:**
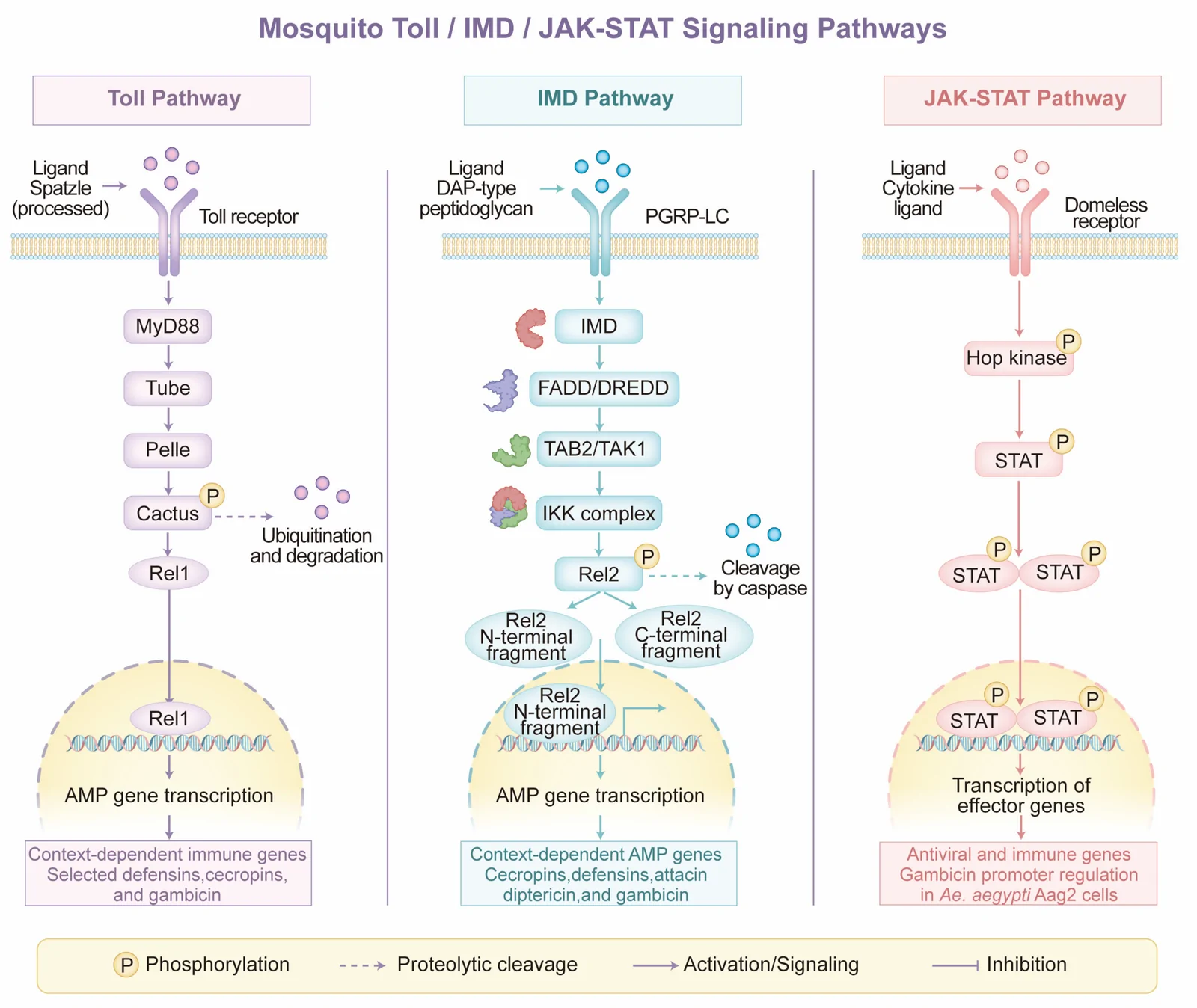
Mosquito Toll, immune deficiency (IMD), and Janus kinase–signal transducer and activator of transcription (JAK–STAT) signaling pathways involved in the regulation of immune effectors. Toll and IMD signaling culminate in the activation of the NF-κB-family transcription factors Rel1 and Rel2, respectively. JAK–STAT represents a distinct cytokine-signaling pathway in which a cytokine-like ligand activates the Domeless receptor, Hop kinase, and STAT. The downstream immune-gene and AMP outputs shown are representative and may vary according to mosquito species, tissue, infection type, and experimental context. In Aedes aegypti Aag2 cells, gambicin promoter activity is regulated combinatorially by Rel1, Rel2, and STAT, whereas AMP induction following Gram-negative bacterial challenge is predominantly associated with IMD signaling [7].

Toll and IMD are the two principal NF-κB-associated pathways that regulate mosquito immune genes, while JAK–STAT is a separate cytokine-signaling pathway (Figure 3) [7,36,38]. Toll signaling proceeds through Spätzle, Toll, MyD88, Tube, Pelle, degradation of Cactus, and nuclear activity of Rel1-family transcription factors. IMD signaling can be initiated by peptidoglycan-recognition proteins such as PGRP-LC and proceeds through IMD, FADD, DREDD, TAK1, the IKK complex, and Rel2. PGRP-LC is therefore a pattern-recognition receptor associated with IMD signaling, not an “IMD receptor.”

Mosquito pathway specificity is not identical to the simplified Drosophila model. In *An. gambiae*, PGRP-LC activates REL2-dependent responses and influences both bacterial homeostasis and Plasmodium infection [39]. In *Ae. aegypti*, forced REL2 expression induces several defensin and cecropin genes and increases resistance to tested Gram-positive and Gram-negative bacteria [40]. Conversely, experiments in Aag2 cells indicate that pathway responsiveness depends on experimental conditions and that the Toll pathway may not respond uniformly to exogenous stimuli [41]. Figure 3 therefore shows conserved architecture and representative outputs, not rigid pathogen-to-pathway assignments.

JAK–STAT contributes to antiviral defense and other immune responses but does not signal through NF-κB. Engineered activation of Dome or Hop in the *Ae. aegypti* fat body reduced infection by several DENV serotypes, while effects differed for other arboviruses [42]. At the AMP level, the clearest mosquito-specific connection is the STAT-binding site in the *Ae. aegypti* gambicin promoter demonstrated in Aag2 cells [7]. It is not appropriate to use the Drosophila genes Vir1 and TotM as representative mosquito AMPs.

### 6.2. Physiological and Environmental Modulation

AMP expression is integrated with development, nutrition, reproduction, and tissue physiology. A mosquito juvenile-hormone-binding protein influences hemocyte development and infection-induced immune responses, linking endocrine state to immune competence [43]. Blood feeding changes microbial load, redox conditions, and gene expression in the gut, so post-blood-meal AMP profiles can reflect multiple inputs rather than a single pathogen signal [4,44].

Environmental stress can also change mosquito fitness and antiviral immunity. Nutritional stress in *Ae. aegypti* altered immune-gene expression and increased susceptibility to DENV in the tested laboratory conditions [45]. Temperature and insecticide-selection studies have reported changes in AMP transcripts, but effects vary among strains, species, developmental histories, and experimental designs. These data support environmental modulation of immunity; they do not yet justify deterministic predictions that a particular climate scenario will increase a specific AMP or reduce transmission in the field.

## 7. Translational Potential and Constraints

Translational strategies can exploit mosquito AMPs in fundamentally different ways: by increasing an endogenous effector within the mosquito, by delivering a heterologous peptide through a transgene or symbiont, or by developing an AMP-inspired therapeutic outside the vector. These routes should not be evaluated by a single standard, because their efficacy endpoints, exposure pathways, ecological footprints, and regulatory requirements differ substantially.

### 7.1. Transgenic and Paratransgenic Delivery

Transgenic expression can place effector peptides at parasite-exposed tissues and developmental windows. Co-expression of cecropin A and defensin A in *Ae. aegypti* reduced *P. gallinaceum* infection in laboratory experiments [34]. Translation to population-level control would additionally require a safe and stable genetic system, acceptable fitness costs, resistance management, and governance appropriate to deliberate mosquito release.

Paratransgenesis modifies mosquito-associated microorganisms to deliver antipathogen effectors. *Asaia* is attractive because transformable strains can colonize mosquito tissues and spread horizontally, vertically, and transstadially in experimental systems [23,46]. Engineered *Asaia* secreting scorpine fusions reduced *P. berghei* oocyst burdens, although recombinant expression imposed bacterial fitness costs and performance depended on the fusion partner [24]. These results establish laboratory proof of concept, not field-ready efficacy.

Environmental release of engineered symbionts raises distinct biosafety and regulatory questions. Required evaluations include host range, persistence outside the target mosquito, genetic stability, horizontal gene transfer, reversibility or recall, effects on resident microbiota and food webs, evolution of parasite resistance, occupational exposure, community engagement, and post-release surveillance [47]. Chromosomal integration and biological containment may reduce some risks compared with mobile plasmids, but they do not eliminate ecological uncertainty. Regulatory requirements will vary by jurisdiction and should be addressed before semi-field or open-release studies.

### 7.2. Wolbachia-Based Control Is Not an AMP-Specific Intervention

Population replacement with wMel-infected *Ae. aegypti* has strong epidemiological evidence for dengue control. In the cluster-randomized AWED trial in Yogyakarta, releases reduced virologically confirmed dengue with a protective efficacy of 77.1% (95% confidence interval, 65.3–84.9%) [48]. This field result validates *Wolbachia* population replacement as a vector-control approach in that setting, but it does not identify AMP induction as the principal mechanism.

Laboratory studies support several nonexclusive mechanisms of *Wolbachia*-mediated pathogen blocking, including immune activation, ROS signaling, altered lipid availability, intracellular competition, and density-dependent effects [32,49]. The relative contribution of these processes differs among *Wolbachia* strains, mosquito backgrounds, tissues, temperatures, and viruses. *Wolbachia* should therefore be discussed as an established or emerging biological-control platform whose relationship to AMPs is mechanistically informative but not singular.

Implementation also requires entomological and epidemiological surveillance, quality control during mass rearing, monitoring of *Wolbachia* frequency and phenotype, assessment of local acceptability, and plans for unexpected ecological or operational outcomes. The WHO is updating its evidence assessment and guidance for *Wolbachia* population replacement, underscoring the importance of context-specific evaluation and governance [50].

### 7.3. Peptide and Drug-Template Development

Mosquito-derived AMPs can serve as templates for anti-infective design, but native peptides are rarely ready-made drugs. Barriers include proteolytic instability, binding to serum or tissue components, salt sensitivity, short half-life, manufacturing cost, delivery to the relevant compartment, immunogenicity, and cytotoxicity. The Anopheles cecropin B study illustrates the trade-off: the full-length peptide had promising in vitro antimalarial activity but also mammalian-cell toxicity and weak in vivo performance, whereas a truncated derivative reduced cytotoxicity and improved efficacy in a mouse model [21].

Rational truncation, residue substitution, cyclization, terminal modification, formulation, and targeted delivery may improve the therapeutic index. Each engineered peptide nevertheless requires quantitative comparison with the parent sequence, including activity, hemolysis, mammalian-cell toxicity, stability, pharmacokinetics, and resistance selection. Evidence from a cultured pathogen or insect model should not be described as clinical potential without these intermediate steps.

## 8. Knowledge Gaps and Research Priorities

### 8.1. Classification and Reproducible Nomenclature

Genome annotations and AMP names should be linked to stable gene identifiers, species and strain, genome assembly, precursor sequence, predicted cleavage site, and mature peptide. This is especially important for attacin-, diptericin-, holotricin-, and other glycine-rich candidates whose names can shift among annotations. Curated cross-species inventories should distinguish biochemical validation, genetic validation, transcript-only evidence, and computational prediction.

### 8.2. Mechanism at Physiological Concentrations

Mechanistic studies should report peptide purity and folding, target strain or parasite stage, buffer composition, salt and serum conditions, concentration, exposure time, and orthogonal endpoints. Membrane leakage, microscopy, electrophysiology, binding assays, and target-specific rescue or resistance experiments can then distinguish primary targets from downstream damage. For claims involving mitochondria, nucleic acids, ribosomes, or redox pathways, target engagement should be demonstrated in the biologically relevant pathogen or mosquito tissue rather than inferred from another peptide or organism.

### 8.3. From Transcript to Functional Peptide In Vivo

Many mosquito studies quantify AMP mRNA, while fewer measure the processed mature peptide at its site of action. Time-resolved peptidomics, targeted mass spectrometry, spatial imaging, and genetic replacement with activity-deficient mutants could clarify when transcription produces a functional peptide concentration. Such approaches are also needed to determine how AMPs shape commensal microbiota without causing pathological dysbiosis [4,44].

### 8.4. Translational Safety, Durability, and Causality

For transgenic or paratransgenic control, efficacy should be evaluated together with containment, genetic stability, horizontal transfer, fitness costs, resistance evolution, and effects on non-target organisms. For *Wolbachia*, mediation analyses and experimentally tractable biomarkers are needed to determine how much pathogen blocking is attributable to AMPs relative to metabolism, intracellular competition, and other immune pathways. Longitudinal field surveillance remains essential because ecological and evolutionary responses may emerge after deployment.

## 9. Conclusions

Mosquito AMPs are a diverse set of endogenous immune effectors whose composition varies among species. Cecropins, defensins, gambicin, attacin, and diptericin are well-supported families in *Ae. aegypti*, while Anopheles and Culex repertoires differ. Scorpine, magainin, and human defensin 5 are heterologous peptides tested in mosquitoes and should not be classified as natural mosquito AMPs.

The strongest mechanistic evidence is peptide-specific. Selected cecropins bind and permeabilize microbial membranes, *An. gambiae* DEF1 shows a defined target spectrum dominated by Gram-positive bacteria, and gambicin has directly measured but target-dependent activity. Evidence for mitochondrial, nucleic-acid, protein-synthesis, and redox mechanisms is narrower than previously stated and must be tied to the exact peptide and assay. Toll and IMD are NF-κB pathways; JAK–STAT is a distinct pathway that can converge on some immune promoters, including gambicin in *Ae. aegypti* Aag2 cells.

Engineered AMP expression, paratransgenesis, *Wolbachia*, and peptide optimization offer complementary translational possibilities, but their evidentiary bases are different. *Wolbachia* field efficacy does not prove AMP-mediated causality, and engineered symbionts require rigorous assessment of biosafety, ecological effects, horizontal gene transfer, regulation, and durability. Progress will depend on quantitative, reproducible studies that connect sequence and expression to mature-peptide concentration, molecular target, in vivo function, and safe deployment.

## Figures and Tables

**Table 1:** Representative endogenous mosquito AMPs and heterologous peptides tested in mosquitoes.

Peptide/Origin	Mosquito or Test System	Length and Structural Characteristics	Target	Quantitative or Functional Result	Evidence/Ref.
*Ae. aegypti* cecropin A2 (endogenous derivative)	Purified peptide; *P. aeruginosa* and other bacteria	36 aa cationic, amphipathic α-helical derivative of cecropin A	Gram-negative bacteria	MIC: 32–64 µg/mL for most clinical *P. aeruginosa* isolates; 2–32 µg/mL for other tested Gram-negative bacteria	Membrane permeability, LPS and DNA binding [11]
*An. gambiae* DEF1 (endogenous)	Recombinant peptide	Approximately 40 aa mature peptide; six conserved cysteines, three disulfide bonds, CSαβ fold	Bacteria and fungi	Most tested Gram-positive bacteria: 0.1–0.75 µM; little activity against Gram-negative bacteria; selected filamentous fungi inhibited	Direct peptide assay [17]
*An. gambiae* gambicin (endogenous)	Purified 61 aa mature peptide	61 aa, 6.8 kDa mature peptide; eight cysteines and four disulfide bridges	Bacteria, fungus, *P. berghei* ookinetes	Killed both Gram classes; morphogenic effect on a filamentous fungus; marginal ookinete lethality	Direct peptide assay [19]
Anopheles cecropin B (endogenous)	Synthetic peptide; cultured blood-stage *P. falciparum*	Cecropin B is a short, cysteine-free, cationic α-helical peptide; full-length sequence tested	Drug-sensitive and drug-resistant *P. falciparum*	IC50: 9.89–12.81 µM; cytotoxicity and weak mouse-model efficacy for full-length peptide	In vitro and mouse study [21]
Scorpine (scorpion; heterologous)	Purified peptide; later engineered *Asaia* delivery	75 aa scorpion-venom peptide with an N-terminal cecropin-like region and a cysteine-rich C-terminal region	*P. berghei* ookinetes and gametes	ED50: 0.7 µM (ookinetes) and 10 µM (gametes); engineered *Asaia* reduced mosquito oocyst burden	Direct/paratransgenic [22,23,24]
Magainin (frog; heterologous)	Injected into infected anopheline mosquitoes	Short, cationic amphipathic α-helical peptide from amphibian skin	Plasmodium sporogonic stages	Aborted normal oocyst development and prevented sporozoite formation in the tested system	Injection study [25]
Human defensin 5 (heterologous)	200 µg/mL microinjection into *An. stephensi*	Cysteine-rich human α-defensin; disulfide-stabilized β-sheet fold	*P. yoelii*	Reduced parasite density; effect weakened after MyD88 silencing	Functional mosquito study [26]

## Data Availability

No new data were created or analyzed in this study. Data sharing is not applicable to this article.
